# Pulmonary Fibrosis in COVID-19 Survivors: Predictive Factors and Risk Reduction Strategies

**DOI:** 10.1155/2020/6175964

**Published:** 2020-08-10

**Authors:** Ademola S. Ojo, Simon A. Balogun, Oyeronke T. Williams, Olusegun S. Ojo

**Affiliations:** ^1^Department of Anatomical Sciences, St. George's University School of Medicine, St. George's, Grenada; ^2^Department of Surgery, Obafemi Awolowo University Teaching Hospital Complex, Ile Ife, Nigeria; ^3^Department of Medicine, Obafemi Awolowo University Teaching Hospital Complex, Ile Ife, Nigeria; ^4^Department of Morbid Anatomy, Obafemi Awolowo University Teaching Hospital Complex, Ile Ife, Nigeria

## Abstract

Although pulmonary fibrosis can occur in the absence of a clear-cut inciting agent, and without a clinically clear initial acute inflammatory phase, it is more commonly associated with severe lung injury. This may be due to respiratory infections, chronic granulomatous diseases, medications, and connective tissue disorders. Pulmonary fibrosis is associated with permanent pulmonary architectural distortion and irreversible lung dysfunction. Available clinical, radiographic, and autopsy data has indicated that pulmonary fibrosis is central to severe acute respiratory distress syndrome (SARS) and MERS pathology, and current evidence suggests that pulmonary fibrosis could also complicate infection by SARS-CoV-2. The aim of this review is to explore the current literature on the pathogenesis of lung injury in COVID-19 infection. We evaluate the evidence in support of the putative risk factors for the development of lung fibrosis in the disease and propose risk mitigation strategies. We conclude that, from the available literature, the predictors of pulmonary fibrosis in COVID-19 infection are advanced age, illness severity, length of ICU stay and mechanical ventilation, smoking and chronic alcoholism. With no proven effective targeted therapy against pulmonary fibrosis, risk reduction measures should be directed at limiting the severity of the disease and protecting the lungs from other incidental injuries.

## 1. Introduction

An outbreak of the novel coronavirus nCoV-19 (SARS-CoV-2), responsible for the coronavirus disease-19 (COVID-19), was first reported in Hubei province, China, on December 31, 2019. It has rapidly spread globally with approximately 3 million confirmed infections and 200,000 deaths within the first four months [1]. Similar to the etiological agents in previous human coronavirus outbreaks (severe acute respiratory syndrome (SARS) and Middle East respiratory syndrome (MERS)), SARS-CoV-2 primarily affects the respiratory system. Clinical, radiographic, and autopsy reports of pulmonary fibrosis were commonplace following SARS and MERS, and current evidence suggests pulmonary fibrosis could complicate infection by SARS-CoV-2 [2]. Pulmonary fibrosis is also a known sequela of severe and/or persistent damage to the lung from other causes such as connective tissue disorders, chronic granulomatous diseases, medications, and respiratory infections [3].

Fibrosis could be viewed as a consequence of a disordered wound healing process and may be directly related to the severity of an inciting event [4, 5]. Various mechanisms of lung injury in COVID-19 have been described, with both viral and immune-mediated mechanisms being implicated [6]. Apart from these, additional factors could predispose individuals to severe lung injury and lead to an increased risk of mortality or pulmonary fibrosis in survivors. In this review, we discuss the pathological mechanisms involved in the development of fibrosis and explore the pathogenesis of lung injury in COVID-19 infection and review reports of pulmonary fibrosis following previous and current human coronavirus outbreaks. In addition, we review the evidence in support of the risk factors for the development of lung fibrosis following COVID-19 infection and putative risk mitigation strategies.

## 2. Methodology

A literature search was conducted to identify relevant articles using PubMed and Cochrane Library up to April 27, 2020, for all studies related to COVID-19, SARS, and MERS, with respect to acute lung injury and pulmonary fibrosis, using a combination of standardized search terms. Cohort studies, case series, cross-sectional studies, and randomized controlled trials were identified and screened for relevance. Articles on the mechanisms of acute lung injury, pulmonary fibrosis, and SARS-CoV-2-induced pulmonary damage, in addition to reports on pulmonary fibrosis following SARS, MERS, and COVID-19 as well as predictors of pulmonary fibrosis, were selected for a full-text review. Additional studies not identified by the database search were identified from citations within the articles.

## 3. Pathogenesis of Pulmonary Fibrosis

An initial phase of lung injury is followed by acute inflammation as well as an attempt at repair [3]. This process can result in the restoration of normal pulmonary architecture, or it may lead to pulmonary fibrosis with architectural distortion and irreversible lung dysfunction. The repair process involves regeneration by native stem cells and connective tissue deposition to replace areas of defect [7]. Alveolar macrophages play a central role in this process by phagocytizing alveolar debris and the production of cytokines and growth factors involved in the repair [8].

The process of repair involves angiogenesis, fibroblast activation, and collagen deposition [9]. In the presence of alveolar exudates, organization occurs which is identified by fibroblastic invasion of the alveoli and transformation into myofibroblasts leading to the deposition of an organizing fibroblastic extracellular matrix (ECM) [9]. Epidermal growth factor (EGF) and transforming growth factor-alpha (TGF-*α*) stimulate proliferation of bronchiolar stem cells to replace damaged alveolar epithelium [10]. Vascular endothelial growth factor (VEGF) and fibroblast growth factor (FGF) stimulate the migration and proliferation of uninjured endothelial cells leading to pulmonary capillary angiogenesis [11].

The degradation of organizing fibroblastic tissue by the fibrinolytic system or remodeling into the interstitium coupled with epithelial and endothelial proliferation is sufficient for the repair process if the basement membranes are intact [4, 12]. However, in severe or persistent injury with damage to the basement membranes, fibroblastic activities persist, converting organizing into fixed and/or progressive fibroblastic tissue [4, 13]. The formation of this scar tissue, either focal or diffuse, results in disorganized alveolar architecture [14]. Excessive deposition of extracellular matrix is therefore central to the process of lung fibrosis. It manifests as an irregular interlobular septal thickening and reticular pattern with traction bronchiectasis on chest CT scan [15]. The process described is typical of pulmonary fibrosis following acute lung injury such as those occurring in severe acute respiratory syndrome (SARS) with diffuse alveolar damage, acute fibrinous, and/or organizing pneumonia [14]. It must be noted, however, that pulmonary fibrosis could occur in the absence of a clear-cut inciting agent and without a clinically obvious initial acute inflammatory phase. This is known as idiopathic pulmonary fibrosis (IPF) or cryptogenic fibrosing alveolitis.

## 4. Pathogenesis of SARS-CoV-2 Infection-Associated Lung Injury

SARS-CoV-2 is an enveloped single-stranded, positive sense RNA virus of the Coronaviridae family [16]. The Orthocoronavirinae subfamily is made up of four members: *α*, *β*, *δ*, and *γ*. The *α* and *β* subgroups are responsible for human infections. SARS-CoV-2 and SARS-CoV and MERS-CoV, two other coronaviruses responsible for previous epidemics, are members of the *β* genera [16, 17]. SARS-CoV-2 shares 79.5% sequence identity with SARS-CoV and 50% identity with MERS-CoV [16, 18]. Similar to SARS-CoV, SARS-CoV-2 binds to human cells using the angiotensin-converting enzyme 2 (ACE2) as a receptor. Meanwhile, MERS-CoV utilizes dipeptidyl peptidase 4 (DPP4) for viral entry [19, 20]. The ACE2 receptor is found in many tissues such as the lung, kidney, heart, and intestines [19, 21].

## 5. The Role of the Immune System in COVID-19-Associated Lung Injury

Following viral entry, the innate immune system recognizes the virus using pathogen-associated molecules, which interact with antigen-presenting cell (APC) receptors to trigger downstream signaling, leading to the release of antimicrobial and inflammatory forces [22]. Toll-like receptor 7 on macrophages is capable of recognizing single-stranded RNA from viruses such as SARS-CoV-2, leading to intracellular signaling via the use of myeloid differentiation factor 88 (MyD88) adaptor protein [23, 24]. This results in the activation of two transcription factors: nuclear factor-kB (NF-kB) which induces the expression of proinflammatory factors and interferon regulatory factors (IRFs) which modulate the expression of interferons (IFNs) [24, 25]. IFNs inhibit viral proliferation, enhance killing of virus-infected cells, and coordinate further immune responses in conjunction with other cytokines [26]. The combined effect of the virus-induced cell injury and inflammatory mediators may be responsible for the lung damage that occurs in COVID-19 [6]. Immune hyperreaction, referred to as cytokine storm due to uncontrolled release of excessive amount of cytokines, has been reported as a major contributor to multiorgan dysfunction in SARS-CoV-2 infection [27]. Elevated levels of cytokines such as IL1-*β*, IL-7, IL-8, IL-9, IL-10, granulocyte-macrophage colony-stimulating factor (GMCSF), IFN-*γ*, monocyte chemoattractant protein (MCP1), and tumor necrosis factor-*α* (TNF-*α*) have been reported in COVID-19 cases, with elevated proinflammatory cytokines correlating with disease severity [28, 29]. Similarly, elevated IL-1, IL-6, IL-8, IL-12, IFN-*γ*, TGF-*β*, and CCL2, CXCL9, CXCL10 chemokines were found in individuals with severe SARS [6].

Simultaneously, the adaptive immune response is also activated. Antigen presentation by APCs through major histocompatibility complex II (MHCII) activates the adaptive immune response via interaction with the CD4+ cell subset of T-cells. This leads to the proliferation and differentiation of CD4+ cells into Th1, Th2, and Th17 subclasses [30]. Th1 response drives inflammation and macrophage activation by secreting IL-2, IFN-*γ*, and TNF. Th2 response produces IL-4, IL-5, IL-10, and IL-13 which mediate antibody production, eosinophilic activation, and immunosuppression [31]. Th17 produces IL-17, a potent stimulator of proinflammatory cytokine secretion [32]. B-cell activation results in the production of IgM, IgA, and IgG. In SARS, IgM is detectable as early as 5-7 days, peaks at 21-30 days, and declines to undetectable levels by 90 days [33]. IgG, which constitutes most of the viral neutralizing antibody, is detectable at 7 days and peaks at about 90 days. It however declines to undetectable levels by 2 years [34]. Lymphopenia has been widely reported in SARS and COVID-19. The mechanism is unknown; however, it is thought to be due to sequestration of lymphocytes in tissues [35]. Coronaviruses are believed to have evolved different mechanisms of immune evasion such as preventing the exposure of ssRNA to pattern recognition receptors as well as inhibiting type 1 interferon production [36, 37].

## 6. Pathogenesis of Alveolar Wall Injury in Coronavirus Infections

The alveolar wall has three components: the alveolar epithelium with its basement membrane, capillary endothelium with a basement membrane, and an interstitium containing the fused basement membranes, fibroblasts, collagen fibrils, elastic fibers, and macrophages [14]. Pulmonary fibrosis usually occurs as a consequence of severe and/or prolonged assault to the lung. It occurs as a result of dysregulation in one or more of the phases of wound healing: injury, inflammation, and repair [3]. The recognized inciting factors include infections, inhaled irritants, chemical injuries and systemic disorders.

Alveolar epithelial damage follows the application of the injurious stimulus, and this results in the release of damage-associated molecular patterns (DAMPs) from injured cells. DAMPs, in addition to pathogen-associated molecular patterns (PAMPs) from microbes, are recognized by alveolar macrophages, leading to a series of downstream transduction, and release of antimicrobial and proinflammatory cytokines, including IL-1 and TNF [22]. TNF as well as IL-1 targets the endothelial cells, resulting in endothelial activation with induction of endothelial leukocyte adhesion molecules (ELAMs) such as ICAM-1, E-selectin, and VCAM-1 which interact with leukocyte surface receptors to mediate leukocyte margination, rolling, and eventual extravasation to the site of injury.

In addition, chemokines from alveolar macrophages such as IL-8 recruit more leukocytes to the site of injury [38]. Damage to the alveolar capillary endothelium from neutrophil adherence to endothelial cells and direct microbe-mediated injury coupled with chemical mediators such as histamine, bradykinin, and leukotrienes increase endothelial permeability. This leads to leakage of fluid into the interstitium and alveolar spaces [3]. Fluid, fibrin, and cellular debris-filled alveolar spaces clinically manifest with respiratory distress. The presence of airspace exudates, alveolar collapse, and interstitial edema show as ground glass opacity, consolidation, and septal thickening upon chest imaging [14].

## 7. Role of Fibroblasts in Pulmonary Fibrosis in Coronavirus Disease

Fibroblasts are the “effector” cells in fibroproliferation. They are mesenchymal cells found in every tissue in the body, playing a vital role in structural support as well as tissue repair following injury. They secrete and regulate the extracellular matrix (ECM) volume [39]. Fibroblasts are found in the alveolar interstitium [14]. Following alveolar injury, fibroblast migration to the site of the injury is stimulated by fibroblast growth factor (FGF), PDGF, TGF-*β*, and chemokines. Fibroblasts proliferate and differentiate into myofibroblasts under the influence of EGF, PDGF, TGF-*β*, and IL-1 [40]. Fibroblasts synthesize collagen, fibronectin, and ECM ground substance. In addition to ECM synthesis, myofibroblasts play an additional role in the inflammatory response by secreting IL-1, IL-6, IL-8, and monocyte chemoattractive protein-1 (MCP-1). In addition, mediators of the repair process such as VEGF and TGF-*β* are secreted by myofibroblasts [41].

Myofibroblasts produce denser but more disorganized ECM than fibroblasts and persist longer at the site of injury. Due to the presence of *α*-smooth muscle actin, they are able to contract irreversibly leading to a spatial reorganization of collagen fibrils, an important feature of fibrogenesis [15]. Other proposed origins of myofibroblasts include pulmonary interstitium pericytes, epithelial mesenchymal transition, and endothelial mesenchymal transition [42]. Epithelial/endothelial to mesenchymal transition occurs via a molecular reprogramming of epithelial/endothelial cells to acquire biological properties of mesenchymal cells [43, 44]. Through this process, alveolar epithelial cells lose their markers such as surfactant proteins, mucin, adhesion molecules (E-cadherin and claudin), and cytoskeletal proteins [44, 45]. These markers are replaced by mesenchymal cell markers such as *α*-smooth muscle actin, vimentin, and fibronectin [45]. This process can be induced by TGF-*β* [46, 47].

In addition to native alveolar fibroblasts, there is evidence supporting the role of fibrocytes in the development of the emerging pulmonary fibrosis [48]. Fibrocytes are circulating hematopoietic derived mesenchymal progenitor cells that produce ECM and have the capacity to differentiate into myofibroblasts [49]. They possess CD34, CD11b, CD18, CD45, and HLA-DR surface markers and express CCR3, CCR5, CCR7, and CXCR4 chemokine receptors [49]. In a study on bleomycin-induced pulmonary fibrosis in mice, Phillips et al. demonstrated that human fibrocytes migrated to the mouse lung in response to CXC chemokine ligand 12 (CXCL 12). In addition, administration of anti-CXCL 12 reduces the population as well as the deposition of collagen in the lungs [50]. Another study by Hashimoto et al. also showed that bone marrow-derived cells play a role in pulmonary fibrogenesis [51]. The elevated level of CXCL12 has been reported in IPF, and it correlates with the degree of lung function [15].

## 8. Role of Cytokines

Macrophage activation during the acute immune response occurs through two pathways known as the classical (M1) and the alternative (M2) pathways. The M1 pathway is initiated by the interaction of PAMPs and DAMPs with macrophage receptors in the innate immune response and stimulation by interferon-y from the T-cell adaptive response. This pathway leads to the production of reactive oxygen species, antimicrobial products, and proinflammatory cytokines responsible for the initial phase of acute inflammation [52]. The M2 pathway is triggered by the effect of IL-4 and IL-13 produced by T-lymphocytes and other cells. This pathway leads to the production of cytokines and growth factors involved in the tissue repair process. It also inhibits the inflammatory process by decreasing the M1 pathway and plays a central role in the formation of scar tissue [53].

Transforming growth factor-*β* (TGF-*β*) is a multifunctional cytokine playing a key role in the process of tissue repair following injury. Important sources of TGF-*β* include *α*-granules of platelets and macrophages in the alternate pathway [54]. Three isoforms of TGF-*β* are found in mammals: TGF-*β* 1, 2, and 3. TGF-*β* stimulates extracellular matrix formation [55]. TGF-*β* 1 is predominantly expressed in the pathogenesis of pulmonary fibrosis. TGF-*β* regulates the formation of all ECM molecules: collagen, fibronectin, elastic fibers, and ground substances [56]. Three TGF-*β* receptors, I, II, and III, are found on the surface of all cells, mediating its effect. The downstream effect is transduced via a serine-threonine kinase pathway(s) leading to transcription of genes involved in ECM formation [54]. TGF-*β* has fibrogenic potential by [1] stimulating fibroblast migration and proliferation, [2] inducing collagen and fibronectin deposition, and [3] inhibiting ECM degradation by matrix metalloproteinases. The stimulation of ECM deposition and inhibition of breakdown is fundamental to excessive accumulation of scar tissue in fibrosis [8, 54].

Platelet-derived growth factor (PDGF) plays a key role in the repair process following tissue injury. The PDGF family has four different polypeptide chains; PDGF-A, PDGF-B, PDGF-C, and PDGF-D. The chains function as dimers linked by disulfide bond [57]. The effect of PDGF is mediated via two similar PDGF tyrosine kinase receptors: PDGFR-*α* and PDGFR-*β*, which can form homo- or heterodimers. The interaction of PDGFs and PDGFRs results in receptor dimerization with autophosphorylation of tyrosine residues between receptors [58]. Further downstream effect is mediated by various molecules including adaptor proteins, phosphatidylinositol-3 kinase, and phospholipase C-*γ* [58]. PDGFs are potent stimuli for the migration and proliferation of fibroblast as well as smooth muscles [59]. It inhibits apoptosis of fibroblasts and other mesenchymal cells [60]. PDGF-C and D have been shown to stimulate angiogenesis via a direct stimulation of vascular stem cells and by upregulation of other angiogenic factors [61]. PDGF has been reported as one of the growth factors playing a key role in pulmonary fibrosis [62]. Bleomycin-induced pulmonary fibrosis is associated with increased expression of PDGF in animal models. Increased PDGF expression has also been documented in IPF and progressive lung fibrosis in rheumatoid arthritis [63, 64].

Epidermal growth factor receptor (EGFR) plays a role in tissue repair following injury. Ligands for this receptor include TNF-*α*, EGF, amphiregulin (AREG), heparin-binding EGF-like growth factor (HB-EGF), epiregulin (EREG), betacellulin (BTC), and epigen (EGN) [65]. EGFR is a membrane-linked tyrosine kinase receptor belonging to the subfamilies of HER1/EGFR/ERBB1, HER2/NEU/ERBB2, HER3/ERBB3, and HER4/ERBB4 [26]. Interaction between ligands and EGFR results in receptor dimerization with autophosphorylation of tyrosine residues in the intracellular domain of the receptor [26]. Further downstream signal transduction activates cellular signaling channels involved in epithelial cell growth, proliferation, and survival [66]. EGFR has been implicated in the pathogenesis of pulmonary fibrosis. Experimental studies show increased TNF-*α* and EGFR expression in bleomycin-lung fibrosis as well as following exposure of the lungs to asbestosis [67, 68]. Doxycycline-regulatable transgenic mouse models of TNF-*α* expression in the lungs show progressive fibrosis following induction of TNF-*α* [69]. In addition, a similar model shows upregulation of AREG and HB-EGF leads to pulmonary pathologies [65].

## 9. Pulmonary Fibrosis Associated with the SARS-CoV and MERS-CoV Infection Outbreaks

SARS and MERS are close “cousins” of SARS-CoV-2 infection, responsible for previous human coronavirus outbreaks. Following the 2003 outbreak of SARS, there were reports of pulmonary dysfunction in convalescent patients. Restrictive lung dysfunction and abnormal carbon monoxide diffusion capacity were commonly reported in patients recovering from SARS. In a study by Muller et al., 5 out of 15 SARS patients who had ground glass opacity (GGO) on high resolution CT scan manifest a combination of irregular interlobular septal thickening and mild traction bronchiectasis 2-17 days after hospital admission [70].

GGO is an area of hazy lung opacity, without obscuring bronchial and vascular markings [8]. GGO can be found in many lung pathologies ranging from benign to malignant conditions; however, the presence of GGO with interlobular septal distortion and traction bronchiectasis are suggestive of pulmonary fibrosis [15]. Wong et al. found GGO with reticulation and irregular interlobular septal thickening in 64 out of 70 (91.4%) patients on CT examination within 48 days after discharge. In that study, follow-up at 3 and 6 months showed the findings were largely unchanged. Most of the patients however had normal pulmonary function tests [70]. Similarly, a follow-up study of 24 patients 5 weeks after discharge found features of pulmonary fibrosis in 62% of the cases [71]. These early imaging findings of pulmonary fibrosis in SARS patients are supported by autopsy reports [72, 73].

Pulmonary dysfunction shows a gradual improvement over months to years in SARS patients [74]. Gradual improvement in lung function results from lung remodeling which is a dynamic repair process following the initial lung injury. This process can restore lung architecture, or it can progress to fixed fibrosis [13]. In a 12-month follow-up study of SARS patients, 85 of 311 patients (27.3%) had within 65 days of discharge developed lung diffusion abnormities (DLCO < 80% predicted) with 67 (21.5%) having lung fibrotic changes. Lung diffusion and fibrosis progressively improved over the duration of study [75]. Ng et al. found restrictive lung dysfunction in 16 of 43 patients at 6 months after discharge [76]. Long-term studies show symptoms persist in SARS patients beyond the early convalescent period in addition to imaging abnormalities. Wu et al. reported 8 out of 11 patients experienced cough, dyspnea, or sputum production 7 years after discharge. GGO with reticulation, suggestive of fibrosis, was found in 10 out of the 11 patients [76]. Similarly, a 15-year follow-up study revealed 10 out of 46 (21.74%) patients had restrictive ventilation dysfunction at three years. However, at 15 years, one had obstructive lung diseases and none had restrictive ventilation dysfunction [77]. Similar to the findings in SARS, pulmonary fibrosis has been reported following MERS. Lung fibrosis developed within 32-240 days after hospital discharge in 33% of MERS patients [78].

## 10. Emerging Clinical Evidence of Pulmonary Fibrosis following SARS-CoV-2 Infection

Acute lung injury, attempt at repair by fibroproliferation, and lung remodeling occur in COVID-19 disease, much like it does in other coronavirus infections. This leads to a potential increase in the risk of pulmonary fibrosis occurring as a sequela of COVID-19 [59]. Fibrotic changes have been found on chest CT scans in COVID-19 patients. In a study of 62 patients by Zhou et al., fibrotic changes were seen in 21 (33.9%) patients, with this finding more likely to occur in advanced-phase disease (8-14 days after the onset of symptoms) than early phase of the disease (≤7 days after the onset of symptoms) [66]. Similarly, Pan et al. reported fibrotic changes on the chest CT scan of 11 out of 63 patients taken during the acute illness [67]. These imaging findings are supported by autopsy reports. Reports on 4 patients who died of COVID-19 pneumonia reveal features of diffuse alveolar damage with areas of consolidation by fibroblastic proliferation and deposition of ECM and fibrin in the alveolar spaces [68]. Similarly, lung explants from 3 patients who had lung transplant for end-stage ARDS show extensive fibrosis of the lungs [79]. The finding of fibrotic changes early in the disease suggests an attempt at repair following pulmonary injury. It is however too early in the process of the disease to determine if this finding would resolve with time or progress to fixed pulmonary fibrosis [80].

## 11. Risk Factors for Pulmonary Fibrosis following SARS-CoV-2 Infection

### 11.1. Age

Lung fibrosis is reported more often in individuals with advanced age. The median age for the diagnosis of idiopathic pulmonary fibrosis is 65 years, and it rarely occurs before 50 years [42]. Similarly, the finding of pulmonary fibrosis correlates with age in SARS. In a follow-up study, advanced age correlated with the risk of developing pulmonary fibrosis at 6 months after discharge (Pearson's correlation coefficient (*r*) = 0.574; *P* = 0.01) [70]. Similarly, older people are more likely to develop pulmonary fibrosis following MERS according to a 230-day follow-up study [78]. The exact reason for this association is unknown; however, older people are more susceptible to both SARS and MERS similar to SARS-CoV-2 infection and are more likely to have severe symptoms [81]. Experimental studies using mouse models reported increased lung fibrosis in older animals compared to younger ones [82]. Additionally, there is an increased resistance of fibroblast and myofibroblasts to apoptosis in older mice, which have been linked to high plasminogen activator inhibitor I (PAI-I), a TGF-*β* effector [7, 82].

### 11.2. Illness Severity

According to the World Health Organization, 80% of SARS-CoV-2 infections are mild, 14% develop severe symptoms, and 6% will become critically ill. Factors associated with increased disease severity include comorbidities such as hypertension, diabetes, and coronary artery disease [83]. Laboratory findings of lymphopenia, leukocytosis, and elevated lactate dehydrogenase (LDH) correlate with increased disease severity [84]. Serum LDH level has been used as a marker of disease severity following acute lung injuries. It is an indicator of pulmonary tissue destruction and correlates with the risk of mortality [70]. The extent of lung injury and inflammatory response correlate with the extent of fibroblastic response required to repair the injury [4, 13]. Peaked LDH level was found to significantly correlate with the risk of pulmonary fibrosis following MERS-CoV infection [78]. Similarly, a follow-up study at 6 months after discharge in SARS patients shows a significant relationship between elevated levels of LDH during acute illness and an increased risk of developing pulmonary fibrosis [70].

### 11.3. Length of ICU Stay and Mechanical Ventilation

ICU care is required in 5-12% of COVID-19 patients, with the criteria for ICU admission varying from one region to another [85, 86]. However, patients with severe conditions, who will likely benefit from ventilatory support, are candidates for ICU admission. In a study of 1300 laboratory-confirmed COVID-19 patients on ICU admission in Lombardy, Italy, 1150 (88.4%) require intubation with mechanical ventilation [87]. While disease severity is closely related to the length of ICU stay, mechanical ventilation poses an additional risk of ventilator-induced lung injury (VILI). VILI is an acute lung injury arising from or exacerbated by mechanical ventilation [88]. The exact incidence is unknown; however, it is associated with increased ICU mortality in ARDS [89]. Abnormalities of pressure or volume settings underlie this injury leading to a release of proinflammatory modulators, worsening acute lung injury, and increased mortality or pulmonary fibrosis in survivors [88]. In a follow-up study of 27 patients who had mechanical ventilation for ARDS, 110–267 days after extubation, 23 (85%) had pulmonary fibrosis with a significant relationship to the duration of pressure-controlled inverse-ratio ventilation (*P* < 0.001) [90].

### 11.4. Smoking

Smoking has been linked to the pathogenesis of various lung diseases such as emphysema, chronic bronchitis, and pulmonary fibrosis. Epidemiological studies show a high incidence of familial and sporadic IPF in smokers when compared to nonsmokers [91]. Smoking is associated with chronic oxidative stress, increased expression of inflammatory cytokines, and interstitial lung fibrosis [92]. The injury associated with smoking continues even after cessation [93]. A systematic review by Vardavas and Nikitara shows that smokers were 1.4 times more likely (RR = 1.4, 95% CI: 0.98–2.00) to have severe symptoms of COVID-19 and 2.4 times more likely to need ICU admission and mechanical ventilation or die compared to nonsmokers (RR = 2.4, 95% CI: 1.43–4.04) [94]. A multivariable logistic analysis by Liu et al. shows the modifiable factor in determining disease progression among factors examined in COVID-19 is current smoking (OR = 14.5, 95% CI: 1.6-25.0) [95].

### 11.5. Chronic Alcoholism

Chronic alcoholism has been cited a predisposing factor for severe respiratory infections [96]. Alcohol abuse is associated with recurrent pneumonia from gastric aspiration. There is also evidence of additional injury to the lung in chronic alcoholism. Clinical and experimental studies show it causes glutathione depletion, chronic oxidative stress, inflammation, and induction of TGF-*β* in the lungs, thereby increasing the risk of acute lung injury and pulmonary fibrosis [97]. It increases the risk of ARDS by three- to fourfold [96]. Similarly, a meta-analysis of 13 studies and 177,674 people shows that alcohol abuse significantly increases the risk of ARDS (OR, 1.89; 95% CI, 1.45-2.48) [98]. By increasing the risk of lung injury and expression of TGF-*β*, a potent fibroproliferative cytokine, chronic alcohol abuse could potentially increase the chance of developing pulmonary fibrosis.

## 12. Strategies for Reducing the Risk of SARS-CoV-2 Infection-Associated Pulmonary Fibrosis

Pulmonary fibrosis is associated with a significant reduction in the quality of life. Two disease-modifying drugs, nintedanib and pirfenidone, have shown promise in clinical trials in slowing down the decline in pulmonary function and have been approved for the treatment of idiopathic pulmonary fibrosis (IPF) [99, 100]. Nintedanib is a tyrosine kinase inhibitor active against growth factor receptors with intrinsic tyrosine kinase activity such as EGFR, VEGFR, and PDGFR. The exact mechanism of action of pirfenidone is not known; however, it is thought to have anti-inflammatory, antioxidant, and antifibrotic properties [42]. Neither of these two drugs has demonstrated significant improvement in symptoms or long-term survival benefit [101]. Lung transplantation is the only treatment option with demonstrable increased survival in selected patients with IPF [30]. However, only a few receive this intervention [42]. Chen et al. reported 3 critically ill patients with COVID-19-related pulmonary fibrosis who had lung transplantation. Two of the patients survived the procedure [79]. Considering the challenges of treating pulmonary fibrosis, there should be greater focus on strategies aimed at reducing the risk among COVID-19 patients. These strategies should be targeted at limiting the factors that perpetuate the cycle of lung injury, inflammatory response, and fibroproliferation.

The viral protease inhibitors: lopinavir/ritonavir, with the RNA polymerase inhibitors: favipiravir and remdesivir, are antiviral agents currently used in many clinical trials in a bid to find an effective therapy against SARS-CoV-2 [102–104]. Similarly, the antimalarials chloroquine/hydroxychloroquine have been used in many clinical trials due to antiviral properties; however, there has been no report confirming clear benefit in COVID-19 [105].

Other medications with antiviral and immunomodulatory properties being considered include nitazoxanide and ivermectin. Although these medications have shown in vitro activity against SARS-CoV-2, further studies are required to determine their efficacy in the treatment of COVID-19 [106]. With cytokine storm due to hyperinflammation being a major factor implicated in lung damage in SARS-CoV-2 infection, the use of immunosuppressive agents has been widely recommended in the treatment of COVID-19 [27]. Immunosuppressive agents that are currently under consideration include the IL-1 receptor blocker anakinra which has demonstrated improved survival benefits in cytokine storm due to sepsis and IL-6 receptor blocker. Tocilizumab is currently registered for a multicenter clinical trial in COVID-19 patients [107, 108]. Despite the common use of corticosteroids, there is little evidence supporting its clinical benefit [27, 109]. The use of convalescent plasma has shown some promise in clinical trials [110, 111]. In addition, mesenchymal stem cells, having multipotent capacity to replace damaged alveolar epithelium, secrete anti-inflammatory factors as well as inhibit fibroproliferation [112] and have been considered in the treatment of ARDS, the leading cause of mortality in COVID-19 disease. The use of mesenchymal stem cells is currently undergoing clinical trials [33].

As the search for an effective therapy against COVID-19 continues, attention should equally be focused on other modifiable factors that could increase the risk of pulmonary fibrosis. The risk of ventilator-induced lung injury should be minimized by the use of protective lung ventilation settings with low tidal volumes and low inspiratory pressures [113]. This has been shown to reduce the relative risk of mortality in ARDS by 30% [113]. In addition, continuous lung injury has been considered a major factor in the development of lung fibrosis. Therefore, patients should be educated prior to discharge on limiting exposure to environmental factors associated with increased lung injury. Smoking cessation has been shown to slow the accelerated pulmonary function decline in chronic obstructive pulmonary disease, suggesting a reduction in pulmonary inflammatory and remodeling process [114]. In measures to reduce particulate inhalation from indoor and outdoor air pollution, an implicated factor in the development of pulmonary fibrosis should be considered [42]. In addition, convalescent patients should be followed up with pulmonary function tests and high-resolution CT scans to monitor changes in lung architecture and function.

## 13. Conclusion

SARS-CoV-2 has continued to spread across the world, infecting millions of individuals in the process. Like previous human coronavirus outbreaks, pulmonary fibrosis has been recognized as a potential sequela among survivors. Virus-induced lung injury, immune response, and attempts at healing are central to the process of fibrogenesis. Predictors of pulmonary fibrosis putatively include advanced age, illness severity, length of ICU stay and mechanical ventilation, smoking, and chronic alcoholism. With no proven effective targeted therapy against pulmonary fibrosis, risk reduction measures should be directed at limiting illness severity and protecting the lung from other incidental injuries.
